# Clinical Characteristics and Audiological Profiles of Patients with Pathogenic Variants of *WFS1*

**DOI:** 10.3390/jcm13164851

**Published:** 2024-08-16

**Authors:** Joonho Jung, Seung Hyun Jang, Dongju Won, Heon Yung Gee, Jae Young Choi, Jinsei Jung

**Affiliations:** 1 Department of Otorhinolaryngology, Yonsei University College of Medicine, Seoul 03722, Republic of Korea; jhjung95@yuhs.ac (J.J.); tkdnsel93@yuhs.ac (S.H.J.); jychoi@yuhs.ac (J.Y.C.); 2 Department of Pharmacology, Graduate School of Medical Science, Brain Korea 21 Project, Yonsei University College of Medicine, Seoul 03722, Republic of Korea; hygee@yuhs.ac; 3 Department of Laboratory Medicine, Yonsei University College of Medicine, Seoul 03722, Republic of Korea; wdjbabo@yuhs.ac

**Keywords:** genetic hearing loss, Wolfram syndrome 1, low-frequency hearing loss, audiologic profile, vestibular profile

## Abstract

**Background:** Mutations in Wolfram syndrome 1 (*WFS1*) cause Wolfram syndrome and autosomal dominant non-syndromic hearing loss DFNA6/14/38. To date, more than 300 pathogenic variants of *WFS1* have been identified. Generally, the audiological phenotype of Wolfram syndrome or DFNA6/14/38 is characterized by low-frequency hearing loss; however, this phenotype is largely variable. Hence, there is a need to better understand the diversity in audiological and vestibular profiles associated with WFS1 variants, as this can have significant implications for diagnosis and management. This study aims to investigate the clinical characteristics, audiological phenotypes, and vestibular function in patients with DFNA6/14/38. **Methods:** Whole-exome or targeted deafness gene panel sequencing was performed to confirm the pathogenic variants in patients with genetic hearing loss. **Results:** We identified nine independent families with affected individuals who carried a heterozygous pathogenic variant of *WFS1*. The onset of hearing loss varied from the first to the fifth decade. On a pure-tone audiogram, hearing loss was symmetrical, and the severity ranged from mild to severe. Notably, either both low-frequency and high-frequency or all-frequency-specific hearing loss was observed. However, hearing loss was non-progressive in all types. In addition, vestibular impairment was identified in patients with DFNA6/14/38, indicating that impaired *WFS1* may also affect the vestibular organs. **Conclusions:** Diverse audiological and vestibular profiles were observed in patients with pathogenic variants of *WFS1.* These findings highlight the importance of comprehensive audiological and vestibular assessments in patients with *WFS1* mutations for accurate diagnosis and management.

## 1. Introduction

Hearing loss is the most common sensorial disorder in humans, with genetic factors accounting for approximately 60–70% of cases [1]. So far, more than 124 deafness genes and 223 deafness-associated genes have been identified [2,3,4]. Among these genes, DFNA6 (DFN = deafness, A = dominant), DFNA14, and DFNA38 are associated with low-frequency non-syndromic hearing loss (LF-NSHL), which is caused by variants of the *Wolfram syndrome 1* (*WFS1*) gene [5,6].

The *WFS1* gene is located on human chromosome 4p16.1 and consists of eight exons. The *WFS1* gene encodes the transmembrane protein, wolframin. Wolframin is an 890-amino-acid protein with an estimated molecular mass of 100 kDa that plays a pivotal role in the regulation of calcium homeostasis within the endoplasmic reticulum (ER) [7,8]. Wolframin is a resident component of the ER with an N_cytosolic_/C_luminal_ orientation in the ER membrane and has a crucial role in the negative regulation of a feedback loop of the ER stress signaling network [9]. Although the physiological role of wolframin is unclear, wolframin is considered to have a role in protein synthesis, trafficking, and regulation of ER stress [10,11,12,13,14]. In addition, the *WFS1* gene negatively regulates a key transcription factor involved in ER stress signaling [15]. *WFS1* mutations are associated with autosomal recessive Wolfram syndrome and the autosomal dominant type of LF-NSHL (DFNA6/14/38) [16]. Wolfram syndrome is a rare, multisystem disorder characterized by diabetes insipidus, insulin-deficient diabetes mellitus, optic atrophy, and hearing loss [17].

The pathomechanism of DFNA6/14/38 and Wolfram syndrome remains elusive. It is suggested that a deficit in wolframin caused by pathogenic variants of *WFS1* elicits an unfolded protein response and ER stress, leading to cellular apoptosis [17]. Given that wolframin is localized in the canalicular reticulum (specialized form of ER), the role of wolframin in the inner ear is possibly associated with ion homeostasis and regulation of ER stress in the ER [18]. 

A wide range of *WFS1* gene variants are distributed throughout the whole gene [19]. However, pathogenic variants are concentrated in exon 8, which is the largest exon [20,21]. In this study, we aimed to investigate the clinical characteristics, audiological phenotypes, and vestibular function in patients with DFNA6/14/38 to provide a comprehensive profile of the impact of *WFS1* variants.

## 2. Materials and Methods

### 2.1. Patient Enrollment

The present study enrolled patients registered in the cohort for genetic hearing loss, namely the Yonsei University Hearing Loss (YUHL) cohort. Patients with hearing loss who also had a family history of hearing loss or voluntarily had undergone genetic testing were included in this cohort. A total of 10 independent families were enrolled in this study. The enrolled patients consisted of five men (50.0%) and five women (50.0%), with an average age of 32.4 ± 19.9 years. This study was approved by the Institutional Review Board of our hospital (approval no. 4-2015-0659). Written informed consent was obtained from all participants.

### 2.2. Evaluation of Hearing Function

Pure-tone audiometry was performed for all enrolled patients and their family members. The pure-tone air (500–4000 Hz) and bone conduction (500–4000 Hz) thresholds were measured using clinical audiometers in a double-walled audio booth. The hearing threshold was calculated as the average threshold at 500, 1000, 2000, and 4000 Hz. Vestibular function tests, including the video head impulse test, caloric test, and vestibular-evoked myogenic potential (VEMP), were performed.

### 2.3. Genetic Analysis 

Genetic testing using next-generation sequencing was performed for individuals and their family members, as previously reported [22,23,24,25,26,27]. Two-track genetic testing was applied; whole-exome sequencing (WES) or deafness gene panel sequencing was used depending on whether payment was covered by their own insurance system. For panel next-generation sequencing, a 207-deafness gene panel was customized as previously described [25]. For WES, the Agilent SureSelect V5 enrichment capture kit (Agilent Technologies, Santa Clara, CA, USA) was used according to the manufacturer’s sample preparation protocol. MiSeq sequencer (Illumina, San Diego, CA, USA) and MiSeq Reagent Kit v2 (300 cycles) were used for massively parallel sequencing. Sanger sequencing was performed for segregation analyses. Variants with a minimum count of five, minimum coverage of 20, and minimum frequency of 20% were called using the “Basic Variant Caller” function in CLC. Variants with minor allele frequencies >0.5% and >0.05% for recessive and dominant hearing loss genes, respectively, in the dbSNP and gnomAD databases were excluded. Genetic diagnoses were confirmed by a board of otolaryngologists and clinical geneticists according to the hearing loss-specified American College of Medical Genetics and Genomics (ACMG) and the Association for Molecular Pathology (AMP) guidelines in the Deafness Variation Database [28]. 

### 2.4. Statistical Analysis

All analyses were conducted using GraphPad Prism v8.0 (GraphPad Software, San Diego, CA, USA).

## 3. Results

### 3.1. Clinical Characteristics of Patients with WFS1 Variants

**Figure 1:**
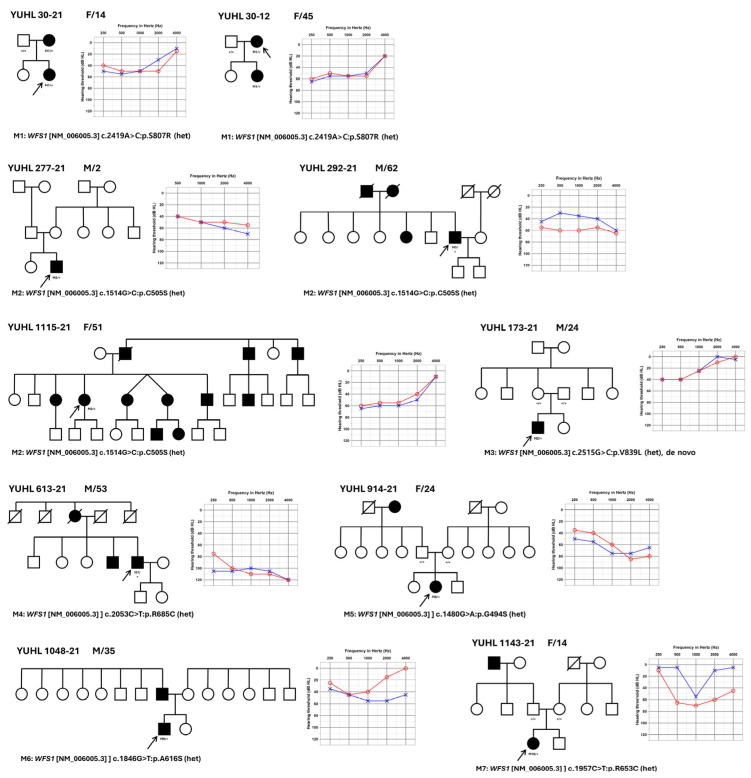
Pedigrees and audiograms of patients with pathogenic variants of *Wolfram syndrome 1* (*WFS1*). The pedigrees and pure-tone audiograms of individuals with pathogenic variants of *WFS1* from 10 independent families are depicted. In the pure-tone audiogram, the red color indicates the auditory thresholds in the right ear and the blue color indicates the auditory thresholds in the left ear.

We analyzed the clinical and genetic characteristics of 10 patients with pathogenic variants of the *WFS1* gene. The patient demographics are shown in Table 1. The pedigree and audiogram of each patient are shown in Figure 1.

The age of onset varied, ranging from the first to the fifth decade. The hearing impairment was bilateral and sensorineural. The average thresholds for the pure-tone audiogram in the right and left ears were 52.8 ± 25.3 dB and 49.5 ± 25.1 dB, respectively, suggesting moderate hearing loss in both ears. Specifically, seven patients (70.0%) had moderate hearing loss (40–69 dB HL), one patient (10.0%) had severe hearing loss (70–89 dB HL), and one patient (10.0%) had profound hearing loss with a hearing threshold >90 dB HL. Although one patient (10.0%) had normal hearing at a pure-tone average, he had hearing loss at low frequencies (40 dB HL at both 250 and 500 Hz) and reported subjective hearing difficulties and vestibular symptoms. Bilateral involvement was observed in all patients (100.0%, *n* = 10). Additionally, four of the ten patients (40.0%) had vestibular symptoms.

Among the 10 affected patients, seven pathogenic variants were identified. Of these, three variants are novel and are reported here for the first time (Table 2), whereas the remaining variants have been previously reported in the literature [29,30,31,32]. All the novel variants were missense (p.C505S, p.R685C, and p.V839L) and have been classified as pathogenic or likely pathogenic according to the ACMG/AMP guidelines.

**Figure 2:**
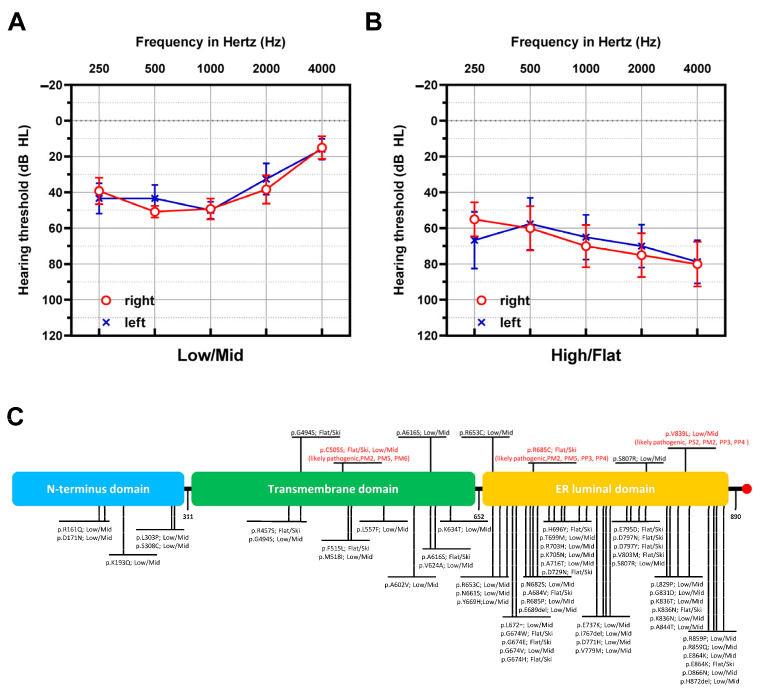
Audiogram configurations in patients with pathogenic variants of *Wolfram syndrome 1* (*WFS1*). (**A**,**B**) The audiogram configuration comprises low/mid (low- and mid-frequency hearing loss, *n* = 6) (**A**) and high/flat (high- and all-frequency hearing loss, *n* = 6) (**B**) types. (**C**) Domain structure of *WFS1* is depicted with variants in *WFS1* and audiological configuration. The variants labeled over the domain figure represent those identified in the present study, whereas those labeled under the domain figure represent those reported previously [34]. Red color refers to the novel variants identified in this study. Low/Mid, low- and mid-frequency hearing loss; Flat/Ski, all-frequency and high-frequency hearing loss; ER, endoplasmic reticulum.

Variants of the *WFS1* gene are known to cause low-frequency sensorineural hearing loss [33]. However, in this study, the audiometry patterns of our patients were diverse. Four patients (40.0%) exhibited flat or ski-slope configurations (Figure 2A), whereas the remaining six patients (60.0%) exhibited low- or mid-frequency hearing loss (Figure 2B). This suggests that the *WFS1* gene can also contribute to high-frequency sensorineural hearing loss. There was no genotype–phenotype correlation in the audiological configuration when we compared all reported variants of the *WFS1* gene (Figure 2C) [34]. Notably, variants in the N-terminal domain were associated with low/mid-frequency hearing loss, whereas those in the transmembrane and ER luminal domains were associated with both flat/ski-slope and low/mid-frequency hearing loss. Therefore, we conclude that the location and type of variants did not affect the pattern of hearing loss, and the factors that should be considered when predicting the patterns of hearing loss remain unclear.

### 3.2. Vestibular Symptoms and Functions in DFNA6/14/38

Vestibular dysfunction has not been extensively studied in patients with DFNA6/14/38. In addition, few studies have disclosed self-reported subjective vestibular symptoms, and vestibular examinations in some patients yielded results within the normal range [35,36]. In this study, vestibular symptoms were present in four patients (40.0%), whereas six patients (60.0%) did not report any vestibular symptoms. Among the four patients with vestibular symptoms, one underwent vestibular examination. Patient YUHS 613-21 with p.R685C exhibited abnormal vestibular examination results; this patient showed decreased gain values in the video head impulse test. The gain values for the anterior, lateral, and posterior canals were 0.79, 0.66, 0.64/0.7, and 0.56/0.62, respectively (right/left). In addition, the patient demonstrated a decreased caloric response in the left ear (canal paresis, 23%). Finally, the VEMP results were abnormal, with no response in either ear. These data indicate that patients with pathogenic variants of *WFS1* commonly experience vestibular symptoms, which may be attributable to abnormal vestibular function.

### 3.3. Progression of Hearing Loss in DFNA6/14/38

**Figure 3:**
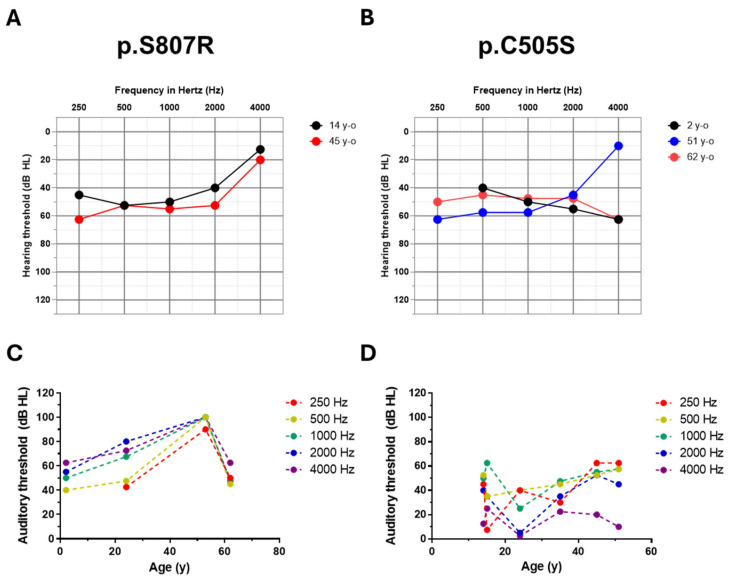
Progression of hearing loss in patients with pathogenic variants of *Wolfram syndrome 1* (*WFS1*). (**A**,**B**) Comparisons of pure-tone audiograms among individuals of different ages. (**A**) Fourteen and forty-five-year-old individuals with p.S807R are compared. (**B**) Two, fifty-one, and sixty-two-year-old patients with p.C505S are compared. (**C**,**D**) Auditory thresholds at individual frequencies are depicted according to the patient ages in high/flat type (**C**) and low/mid type (**D**) of hearing loss.

Providing patients with information regarding the progression of hearing loss is important during genetic counseling. It is not well known whether hearing loss is progressive or the extent to which hearing impairment worsens over one’s lifespan. In this study, we compared the severity of hearing loss among individuals with the same *WFS1* variants. The hearing levels in patients with p.S807R and p.C505S mutations were rarely progressive (Figure 3A,B). The severity of hearing loss in patients with both p.S807R and p.C505S mutations was moderate. Notably, a 62-year-old patient with the p.C505S variant of *WFS1* exhibited a similar level of hearing loss as a two-year-old patient with the same variant. Additionally, there was a tendency for hearing thresholds at all frequencies to not change consistently with age (Figure 3C,D). The non-progressive and stable nature of hearing loss does not differ between low/mid-frequency and high/flat-frequency audiogram configurations. Therefore, we believe that hearing loss in patients with DFNA6/14/38 is not progressive and remains at a mild to moderate level.

## 4. Discussion

We investigated the clinical characteristics of the audiological and vestibular phenotypes in patients with pathogenic variants of the *WFS1* gene. Ten patients with pathogenic variants of *WFS1* exhibited non-syndromic hearing loss with an autosomal dominant inheritance pattern, consistent with DFNA6/14/38. Although the canonical pattern of low-frequency hearing loss was found in only 60% of the patients, the remaining 40% demonstrated hearing loss at high frequencies or across all frequencies. Notably, approximately half of the patients experienced vestibular imbalance, and one patient exhibited impaired vestibular function. Hearing loss was not progressive, and its severity was generally mild to moderate.

More than 250 pathogenic variants of the *WFS1* gene have been identified worldwide (http://www.hgmd.cf.ac.uk/ac/index.php, accessed on 1 May 2024). Variants of the *WFS1* gene are responsible for both Wolfram syndrome and autosomal dominant non-syndromic hearing loss (DFNA6/14/38) [5,37,38]. Wolfram syndrome is an autosomal recessive disorder characterized by diabetes mellitus, diabetes insipidus, optic atrophy, and hearing loss. In non-syndromic hearing loss, DFNA6/14/38 leads to LF-NSHL [33,37,39]. Although variants associated with Wolfram syndrome can be nonsense, frameshift, or missense, those associated with DFNA6/14/38 are predominantly missense, with the exception of one frameshift variant that leads to a truncated wolframin lacking the ER luminal domain (p.Phe515LeufsTer28) [34]. This finding indicates that variants linked to Wolfram syndrome are loss-of-function mutations, whereas variants linked to DFNA6/14/38 are likely gain-of-function mutations (https://omim.org/, accessed on 1 May 2024). Regarding the pathogenesis of DFNA6/14/38, a dominant-negative effect is less likely because such effects are expected to cause Wolfram syndrome. Instead, variants associated with DFNA6/14/38 lead to non-syndromic hearing loss without additional features of optic atrophy, diabetes mellitus, or diabetes insipidus.

According to the literature and the summarized variant map in Figure 2C, the hotspot region for mutations in the *WFS1* gene is located in the ER luminal domain [31,34]. Specifically, the majority of variants associated with LF-NSHL are missense mutations in exon 8. Mutations responsible for LF-NSHL do not inactivate *WFS1*. Although the physiological role of *WFS1* in the inner ear remains unknown, variants in the ER luminal domain cause misfolding of wolframin, leading to protein instability, ER stress, and cellular apoptosis [40,41]. However, it remains unclear why the cochlear apical turns responsible for detecting lower frequencies are more vulnerable to cellular ER stress. In addition, the specific variants that are more strongly associated with low-frequency hearing deterioration remain unknown. Generally, hair cells in the basal turn are more susceptible to ER stress and are prone to damage from chronic noise exposure [42]. Nevertheless, several genetic diseases, including DFNA6/14/38 and DFNA1, have been associated with low-frequency hearing loss. Future studies investigating the biological connection between *WFS1* linked to DFNA6/14/38 and DIAPH1 linked to DFNA1 would be particularly interesting.

It is also worth noting that hearing loss in patients with DFNA6/14/38 was not progressive, although there was significant variability in the severity of hearing loss. Specifically, the severity of hearing loss did not exceed 60 dB in patients with low/mid-frequency hearing loss. By contrast, the hearing threshold tended to be relatively higher in cases with flat or ski-slope audiogram configurations than in those with low/mid-frequency hearing loss (Figure 3C,D). The difference in the severity of hearing loss depending on the audiological configuration suggests that variants leading to flat- or ski-slope-type hearing loss may be more likely to cause a more severe degree of hearing loss than that of those leading to low/mid-frequency hearing loss. These findings should be considered when providing genetic counseling to patients.

Little is known regarding vestibular function and symptoms in patients with DFNA6/14/38. Vestibular dysfunction in DFNA6/14/38 has not been extensively evaluated. A recent report identified mild vestibular impairments in patients with DFNA6/14/38, specifically otolith dysfunction in patients with the p.P838S variant, although this finding may be incidental [35]. In the present study, 40% of patients reported subjective vestibular impairment symptoms, and one patient (YUHL613-21, p.R685C) exhibited decreased gains in both the video head impulse test and the VEMP test, indicating impaired otolith function, which is consistent with findings in a previous report. Notably, two variants associated with abnormal VEMP results are located in the ER luminal domain. However, further case analyses are needed to confirm whether variants in the ER luminal domain affect vestibular function.

In conclusion, LF-NSHL is typically the representative audiological feature of DFNA6/14/38. However, flat or high-frequency types of hearing loss are also frequently observed. Therefore, more caution should be exercised when ruling out the possibility of DFNA6/14/38 in cases of flat or high-frequency hearing loss. Furthermore, given that vestibular dysfunction can also be present in DFNA6/14/38, vestibular function tests should be considered for evaluating vestibular function in patients with *WFS1* variants. A comprehensive evaluation of both audiological and vestibular functions is therefore necessary for patients with variants of *WFS1*.

## Figures and Tables

**Table 1:** Demographics of the enrolled patients.

Characteristics	Value (*n* = 10)
Age (yr)	32.4 ± 19.9
Sex	
Male	5 (50)
Female	5 (50)
Age of onset	
1st decade	4 (40.0)
2nd decade	3 (30.0)
3rd decade	1 (10.0)
4th decade	1 (10.0)
5th decade	1 (10.0)
Side	
Both	10 (100.0)
Pure tone average (dB HL)	
Right	52.8 ± 25.3
Left	49.5 ± 25.1
Hearing loss severity	
Normal (0–25 dB HL)	1 (10.0)
Mild (25–39 dB HL)	0 (0.0)
Moderate (40–69 dB HL)	7 (70.0)
Severe (70–89 dB HL)	1 (10.0)
Profound (>90 dB HL)	1 (10.0)
Pure tone audiometry pattern	
Low/Mid	6 (60.0)
Flat/ski	4 (40.0)
Vestibular Symptoms	
Yes	4 (40.0)
No	6 (60.0)

**Table 2:** Genetic variants of patients.

Gene Symbol	Individual	Age	Sex	Nucleotide Change	Amino Acid Change	Zygosity	gnomAD (EAS)	SIFT	Mutation Taster	PhyloP	GERP++	REVEL	CADD Phred	ACMG/AMP Guideline
*WFS1*	YUHL 30-21	14	F	c.2419A>C	p.Ser807Arg	Het	Absent	Damaging	Disease Causing	Conserved	Conserved	0.521	25.6	[29]
*WFS1*	YUHL 173-21	24	M	c.2515G>C	p.Val839Leu	Het	Absent	Deleterious	Disease Causing	Conserved	Conserved	0.622	24.2	Likely pathogenic (PS2, PM2, PP3, PP4)
*WFS1*	YUHL 277-21 YUHL 292-21 YUHL 1115-21	2 62 51	M M F	c.1514G>C	p.Cys505Ser	Het	0.000381	Tolerated	Disease Causing	Conserved	Conserved	0.731	16.89	Likely pathogenic (PM2, PM5, PM6)
*WFS1*	YUHL 613-21	53	M	c.2053C>T	p.Arg685Cys	Het	0.000555	Damaging	Disease Causing	Non-conserved	Conserved	0.797	32	Likely pathogenic (PM2, PM5, PP3, PP4)
*WFS1*	YUHL 914-21	24	F	c.1480G>A	p.Gly494Ser	Het	0.000401	Tolerated	Disease Causing	Conserved	Conserved	0.862	23.6	[30]
*WFS1*	YUHL 1048-21	35	M	c.1846G>T	p.Ala616Ser	Het	0.000163	Tolerated	Polymorphism	Conserved	Conserved	0.570	13.98	[31]
*WFS1*	YUHL 1143-21	14	F	c.1957C>T	p.Arg653Cys	Het	0.000551	Damaging	Disease Causing	Non-conserved	Conserved	0.817	32	[32]

*WFS1*, Wolfram syndrome 1.

## Data Availability

Data are contained within the article.
